# Investigating the inflammatory mechanism of notoginsenoside R1 in Diabetic nephropathy via ITGB8 based on network pharmacology and experimental validation

**DOI:** 10.1186/s10020-024-01055-8

**Published:** 2024-12-26

**Authors:** ChangYan Li, Chen Geng, JiangMing Wang, Luyao Shi, JingYuan Ma, Zhang Liang, WenXing Fan

**Affiliations:** 1 Department of Nephrology, First Affiliated Hospital, Kunming Medical University, Kunming, Yunnan Province China; 2 Yunnan Key Laboratory of Laboratory Medicine, First Affiliated Hospital, Kunming Medical University, Kunming, Yunnan Province China; 3 Department of Science and Technology, Kunming Medical University, Kunming, Yunnan Province China; 4 Yunnan Province Clinical Research Center for Laboratory Medicine, First Affiliated Hospital, Kunming Medical University, Kunming, Yunnan Province China; 5 Department of Nephrology, First Affiliated Hospital of Kunming Medical University, No.295 Xichang Road, Kunming, 650032 Yunnan Province China

**Keywords:** NGR1, Network pharmacology, Inflammatory injury, ITGB8, Diabetic nephropathy

## Abstract

**Background:**

Diabetes often causes diabetic nephropathy (DN), a serious long-term complication. It is characterized by chronic proteinuria, hypertension, and kidney function decline, can progress to end-stage renal disease, lowering patients’ quality of life and lifespan. Inflammation and apoptosis are key to DN development. Network pharmacology, clinical correlation, and basic experimental validation to find out how NGR1 might work to reduce inflammation in DN treatment. The study aims to improve DN treatment with new findings.

**Methods:**

To determine how NGR1 treats DN, this study used network pharmacology, clinical correlation, and basic experimental validation. Three methods were used to predict NGR1 drug targets: ChEMBL, SuperPred, and Swiss Target Prediction. Drug targets are linked to diseases by molecular docking. A clinical correlation analysis using the Nephroseq Classic (V4) database looked at the strong link between medication targets and the development, progression, and renal function of DN. Additional research showed that NGR1 reduces high blood sugar-induced podocyte inflammation.

**Results:**

The integrin subunit beta 8 (ITGB8) protein is a potential NGR1 therapeutic target for DN. It may be linked to inflammatory proteins like caspase 3 and IL-18. Validation of the molecular docking showed that SER-407, ALA-22, Ala-343, and TYR-406 form hydrogen bonds with NGR1 and ITGB8. These interactions represent pharmacodynamic targets. Clinical correlation showed that DN patients had significantly lower ITGB8 expression levels than healthy individuals. Between 50 and 80 years old, DN patients’ ITGB8 expression levels decreased. ITGB8 expression was lowest in renal function conditions, with eGFR values of 15–29 ml/min/1.73 m2. In the db/db mouse model, downregulation of ITGB8 expression in renal tissue was associated with renal inflammatory damage. The hyperglycemic group had significantly lower levels of nephrin and caspase-3 protein, but higher levels of cleaved caspase-1 protein. Giving NGR1 in different amounts (1, 3, 10, and 30 µM) greatly decreased the expression of caspase3, stopped the expression of cleaved caspase1, and lowered the damage caused by NLRP3 in podocytes.

**Conclusion:**

We identified several NGR1 pharmacological targets and found that the ITGB8 protein is a key drug target linked to inflammation and DN. ITGB8 is critical for DN development and can help to reduce high blood sugar-induced podocyte inflammation.

Diabetes continues to be the primary cause of kidney failure or end-stage kidney disease (ESKD) in adults. Among Asian populations, approximately 68% of patients with chronic kidney disease experience a decline in glomerular filtration function and elevated protein levels, with an incidence rate of 17% (Wang et al. 2023). Additionally, 30–40% of patients with diabetes mellitus will develop renal lesions (Rossing et al. 2018). DN is a prevalent complication of diabetes characterized by chronic microvascular damage. Failure to intervene at an early stage will result in more severe complications and negative outcomes for patients (Hua 2020). Various factors influence the occurrence and progression of DN, such as hemodynamic disorder, insulin resistance, glucose and lipid metabolism disorder, inflammation, and hypoxia (Samsu 2021). Nevertheless, the precise mechanism by which DN causes disease has yet to be established. Oxidative stress and chronic inflammation are now recognized as significant factors in the development of DN (Leszek et al. 2016). Blocking the renin-angiotensin-aldosterone system, controlling blood sugar levels, and encouraging weight loss, might not always work well in many clinical settings (Malek et al. 2021). Traditional Chinese medicine (TCM) offers a diverse range of resources with minimal toxicity and side effects. It also has the advantage of targeting multiple drug targets, making it a potentially advantageous choice as a primary or adjunct medication (Wang et al. 2014). Researchers have found that some TCMs, such as genistein and astragalus polysaccharides, have been used to treat DN and can protect the kidneys by blocking the TLR4/NF-κB signaling pathway (Guo et al. 2023).

*Panax notoginseng*, a traditional Chinese botanical remedy belonging to the Araliaceae family, has been extensively used for millennia in Eastern nations. The pharmacological properties of *Panax notoginseng* are well-documented, with a novel phytoestrogen, notoginsenoside R1 (NGR1), identified as a key active component responsible for its therapeutic effects (Xu et al. 2018). Furthermore, *Panax notoginseng *is known for its positive impact on blood circulation, alleviating pain, and removing blood stasis, and has been widely used in the prevention and treatment of microcirculatory disorders. Additionally, it exhibits anti-inflammatory properties, reducing inflammatory responses in both human and animal models, though it may exacerbate symptoms of experimentally induced disseminated intravascular coagulation. These diverse pharmacological effects of *Panax notoginseng* provide a critical foundation for investigating the therapeutic potential of NGR1, particularly in the context of DN (Tang et al. 2023; Tan et al. 2021; Liu et al. 2020).

Previous studies have provided significant insights into the pathophysiology of DN and the therapeutic potential of NGR1. For example, Zhang et al. established an experimental model using db/db mice and HK-2 cells exposed to advanced glycation end products (AGEs) (Zhang et al. 2019). In *vivo *findings demonstrated that NGR1 treatment increased blood lipids, β2-microglobulin, serum creatinine, and urea nitrogen levels in db/db mice. Despite these changes, NGR1 mitigated histological abnormalities in the kidney, evidenced by reduced glomerular volume and fibrosis in diabetic kidneys. Furthermore, in vitro experiments showed that NGR1 reduced AGE-induced mitochondrial damage, limited the increase in reactive oxygen species (ROS), and decreased apoptosis in HK-2 cells. Similarly, Li et al. (Li et al. 2023). adopted a transcriptome-wide approach to identify differential genes in DN and leveraged the Spearman algorithm and Swiss target prediction to predict potential drug targets for NGR1. Their cellular experiments confirmed that NGR1 improved podocyte apoptosis under high glucose conditions by regulating the expression of FGF1 and VEGFA.

Building on these foundational studies, our research aimed to further elucidate the therapeutic potential of NGR1 in DN by identifying and validating its drug targets, particularly focusing on inflammatory targets such as ITGB8. We conducted a comprehensive analysis of pharmacological databases, followed by molecular docking and molecular dynamics simulations, to confirm the interaction between NGR1 and ITGB8. Our animal and cellular experiments revealed that ITGB8 expression is downregulated in DN patients, and that NGR1 can upregulate ITGB8 expression, potentially mitigating inflammatory injury in podocytes.

## Materials and methods

### Access to drug targets and DN

The target information was annotated in the CTD (http://ctdbase.org/) and ChEMBL (https://www.ebi.ac.uk/chembl/) database with the key word “Notoginsenoside R1”. GSE30122 was obtained from the NCBI-GEO (https://www.ncbi.nlm.nih.gov/geo/) database. Difference of gene expression data was analyzed based on the “Limma” R package under the conditions of Logfoldchange = 1, adjust *P* = 0.05, and DN-related differential genes were obtained. In addition, the genes related to cell scorching were collected from the MSIGDB(http://www.gsea-msigdb.org/gsea/msigdb) and Gene Ontologyhttps://geneontology.org/Database. Core genes were analyzed for protein-protein interaction using the STRINGhttps://string-db.org/database, and Cytoscape 3.7.2 was used to optimize the results.

### Data acquisition and differential gene identification

In this study, we aimed to identify potential inflammation-related targets in DN. To achieve this, we obtained three key gene expression datasets from the GEO database (Na et al. 2015): GSE30122, GSE30528, and GSE142153. These datasets include transcriptome data from DN patients and control groups. We utilized the Limma package for differential expression analysis. Initially, each dataset was normalized to eliminate technical noise and batch effects, ensuring data consistency and comparability. Using the Limma package, we performed differential expression analysis on the preprocessed transcriptome data. The filtering criteria were set to a *P*-value of less than 0.05 (*P* < 0.05) and an absolute log fold change greater than or equal to 2 (|LogFC|≥2), to ensure that the identified genes exhibit significant expression changes. These genes are considered to play crucial roles in the onset and progression of DN.

To further investigate the inflammation-related mechanisms in DN, we obtained the “HALLMARK_INFLAMMATORY_RESPONSE” gene set from the GSVA (Gene Set Variation Analysis) database. This gene set includes key proteins and genes associated with the inflammatory response, aiding in the identification of potential inflammation-related targets in DN. We performed a Venn diagram analysis to intersect the differentially expressed genes from the differential expression analysis with the “HALLMARK_INFLAMMATORY_RESPONSE” gene set. The intersecting genes are both differentially expressed in DN and related to the inflammatory response, indicating their potential role in the inflammatory mechanisms of DN. Through this Venn diagram analysis, we can identify potential inflammation-related targets in DN. The expression changes of these genes may reflect the pathophysiological characteristics of DN, particularly the regulation of the inflammatory response. Further research will focus on these intersecting genes to explore their specific functions and potential therapeutic value in DN.

### Molecular docking procedure

Network pharmacological methods identified ITGB8 as a potential drug target for DN. To further screen the pharmacodynamic interactions between drugs and disease, we employed molecular docking techniques. The process involved the following steps:

Using network pharmacology, we identified ITGB8 as a key target for DN. The identification process involved analyzing the interaction network between known DN-associated proteins and potential therapeutic compounds. The 3D structure of the ITGB8 protein was obtained from the Protein Data Bank (PDB) and protein structure was cleaned by removing water molecules, heteroatoms, and any bound ligands. Protonation states of ionizable residues were adjusted to reflect physiological pH (7.4).

Energy Minimization: The protein structure was subjected to energy minimization using software such as GROMACS or Chimera to relieve any steric clashes and optimize the geometry. The 3D structures of the potential therapeutic compounds were obtained from databases like PubChem, and the protonation states of the ligands were adjusted according to physiological pH using software such as OpenBabel.

Energy Minimization: The ligands were also subjected to energy minimization to optimize their geometry. The active site of the ITGB8 protein where the ligand is expected to bind was identified. This could be based on the binding site of a co-crystallized ligand or predicted using tools like CASTp or SiteMap. A grid box was generated around the active site using docking software such as AutoDock Vina. The grid box size was adjusted to encompass the entire binding site and allow for flexibility in ligand binding. The docking scores, which indicate the binding affinity of the ligands to the protein, were analyzed. Lower scores typically indicate stronger binding. The best docking poses were selected based on binding affinity scores. Key interactions, such as hydrogen bonds, hydrophobic interactions, and π-π stacking, were visualized and analyzed using tools like PyMOL or Discovery Studio. Specific hydrogen bond interactions between pharmacodynamic targets were identified. For example, hydrogen bonds between SER-407 and ALA-22, and between ALA-343 and TYR-406, were highlighted. By following this detailed molecular docking procedure, we were able to predict the binding interactions between the candidate ligands and the ITGB8 protein, providing insights into their potential efficacy and mechanism of action in the treatment of DN.

### Molecular dynamics simulation section

The molecular dynamics simulation section has been expanded to include detailed validation procedures, referencing the methodology of Zhao et al.(Zhao et al. 2024) with slight modifications. Using AMBER 18’s Antechamber (Wang et al. 2000), the Generalized AMBER force field (GAFF) was employed to generate the ligand topology file. Subsequently, the Amber99sb-ildn force field within the GROMACS 19.5 software package (https://manual.gromacs.org/) was utilized to construct the protein topology file, which was then combined with the ligand topology file to form the protein-ligand complex topology file.

Molecular dynamics simulations (MDs) of the protein-ligand complex were conducted in GROMACS 19.5, with TIP3P as the solvent in a cubic box under periodic boundary conditions. To neutralize the charge, 48 Na + and 45 Cl- ions were added as necessary. The system was initially optimized using the steepest descent method until the energy reached < 1000.0 kJ/mol/nm. Following energy optimization, a 1 ns NVT (constant number of particles, volume, and temperature) equilibration and a 2 ns NPT (constant number of particles, pressure, and temperature) equilibration were performed to maintain the system at a constant temperature of 310.15 K and pressure of 0.1 MPa. Subsequently, a 100 ns MDs simulation was carried out, accelerated by an NVIDIA GeForce RTX 3080 GPU.

The XTC files of the protein-ligand complex from 80 to 100 ns were extracted, and the binding free energy between the ligand and receptor was calculated using the Molecular Mechanics Generalized Born Surface Area (MM/GBSA) method implemented in gmx_MMPBSA (Valdés-Tresanco et al. 2021). Additionally, the 3D conformations of the protein-ligand complex were extracted every 10 ns using gmx trjconv. Further analysis was performed using GMX commands to extract the Root Mean Square Deviation (RMSD), Radius of Gyration (Rg), Solvent Accessible Surface Area (SASA), and the number of hydrogen bonds between the ligand and receptor.

### Model building

#### *Vitro* model

Human renal podocytes were purchased from Shanghai Qincheng Biotechnology Co., Ltd. with the product number QC822. The cells were cultured in a 37 °C incubator containing 5% CO_2_, using a medium composed of 89% McCoy’5 A, 1% double antibiotics, and 10% fetal bovine serum (FBS). A podocyte injury model was established by added D-Glucose anhydrous to the medium to make it contain high glucose (30mM), and used the prepared medium to culture cells with a concentration of 30 mM for 24 h to construct a high glucose model. Concurrently, a control group, a high glucose intervention group (30 mM, treated for 24 h), and an NGR1 group (1, 3, 10, 30 µM, with drug intervention administered when cell density reached approximately 70-80% for 12 h) were established to investigate the mechanisms of NF-κB-induced inflammation. Additionally, NLRP3 inhibitor (CY09 1μM) with the product number 1073612-91-5 and a purity of ≥ 98.0% was administered to interfere with the process. The inhibitor was added 30 min prior to modeling. Furthermore, a hyperosmolar mannitol control group (30 mM, treated for 24 h) was included.

#### Animals

The db/db mice are type 2 diabetic mice with point mutations in the Leptin receptor. They develop obvious obesity and hyperglycemia as well as other diabetic symptoms 6 weeks after birth. These symptoms are most pronounced between 8 and 12 weeks, and DN and other complications may occur. Six 7-week-old SPF male C57BLKS (BKS) homozygous Lepr^-/-^(db/db) mice weighing 31.5–35.8 g were purchased from Changzhou Cavens Experimental Animal Co., Ltd. Three C57BL/6J background heterozygous (db/m) mice weighing 23.3–26.6 g were also procured, with strain number C000110. The animals were housed in the SPF laboratory of the Department of Animal Science, Kunming Medical University, at a constant temperature of 25 ± 2 °C and relative humidity of 55 ± 5%, with a 12-hour light/dark cycle. All mice were fed adaptively for 1 week before the experiment. The animals were kept in individually ventilated cages (6 mice per cage) with a 12-hour light/dark cycle. All animals had free access to standard rodent pellet feed and drinking water and were allowed to adapt to the environment for a week. Three 8-week-old db/db mice were selected and injected with NGR1 (30 mg/kg) daily via intraperitoneal injection for 10 consecutive days before sampling. The remaining 3 db/db mice served as the model group.

#### The NF-κB detection reagent

NF-κB nuclear translocation experiment was purchased from Beyotime Biotechnology Institute with the product number: SN368. The brief procedure involves adding the immunostaining blocking solution for 1 h at room temperature, aspirating the blocking solution, and then incubating with the NF-κB p65 antibody overnight at 4 °C. Subsequently, anti-rabbit Cy3 is added and incubated for 1 h at room temperature. Next, the nuclear staining solution (DAPI) is added for approximately 5 min at room temperature, followed by adding the anti-fluorescence quenching mounting medium. After sealing with a coverslip, the slides are observed under a fluorescence microscope. NF-κB staining appears as red fluorescence, while DAPI staining of the cell nuclei appears as blue fluorescence.

#### Trypan blue staining to detect apoptosis

About 5 × 107 cells were immobilized in 4% paraformaldehyde at room temperature for 10 min. The 50–100 mL cell suspension was dripped and dried. Wash with PBS twice for 5 min each time. Reaction at room temperature for 5 min. Wash with PBS twice for 5 min each time. Drain the tissue around the slide carefully with filter paper. Immediately add two drops of TDT enzyme buffer to the slide and leave it at room temperature for 1–5 min. Next, add 54 mL TDT enzyme reaction to the section, and the reaction was carried out in a wet box at 37 °C for 1 h -note: negative staining control plus reaction liquid with (TDT enzyme). The slides were placed in the dye VAT and added with the washing and terminating reaction buffer preheated to 37 °C. The slides were at 37 °C for 30 min, then gently lifted and lowered every 10 min to stir the liquid slightly. The tissue sections with PBS 3 times, 5 min after each time, two drops of Peroxidase labeled anti-di Gaucín antibody was added directly onto the sections, carried out at room temperature in a wet box for 30 min. Wash with PBS 4 times for 5 min each time. New 0.05% DAB solution was added directly onto the tissue sections, developing the color at room temperature for 3–6 min. Wash four times with distilled water, the first three times 1 min each time, the last one time 5 min. Put Methyl green re-dyed at room temperature for 10 min. Rinse with distilled water three times, lift and lower the slide ten times the first two times, and leave the slide for 30 s the last time. Wash three times with 100% butanol in the same way. The xylene was dehydrated three times, each time for 2 min. After sealing and drying, observed and recorded under the optical microscope.

#### Cytotoxicity test (CCK8 method)

In short, high glucose and NGR1 interfere with human renal podocytes and then add 10 µL of CCK-8 solution (Kyushu, Japan) to check cell viability. Put the sample incubating at 37 °C for 2 h, and the absorbance tested at 450 nm by Bio-Rad (Hercules, CA).

#### Immunofluorescence

Inoculating Human Primary podocytes (HPCC) into a 48-well plate, the standard control group was cultured in a McCoy’5 A medium. The model group and high glucose (30mM) were washed 3 times with PBS (GIBCO, 8121388us), then 1004 ΜL polyformaldehyde (Beyotime, P0099, China) was added into each hole. The cells were washed 3 times with 0.5% Triton X100 (Beyotime, ST795, China) and soaked at room temperature for 30 min to make the cell membrane permeable. Using PBS 3 times, meanwhile, sealed the cells with 5% PBS dilution of bovine serum (OriCell, FBSST-01033-500, US). Goat anti-rabbit IgG Alexa Fluor 594 (ZENBIO, 550043,1:1000, China) was washed 3 times with PBS at room temperature for 30 min, 10 ΜL DAPI (Sigma, SLBS2101V, Germany). Look at the slide under the Fluorescence microscope.

#### Western blotting

RAPI Lysate extracted the total protein, and the BCA kit detected the protein concentration. Besides, using 10% SDS-PAGE electrophoresis to isolate the proteins. Then the target bands were transferred to PVDF membrane by semi-dry method, and then sealed 5% skimmed milk powder at room temperature for 1 h. What’s more, the diluted and related primary antibody was added, incubated overnight at 4 °C, and added secondary antibody. After incubating at room temperature for 2 h, and then carrying ECL out dyeing, Gel Imager collected the Image, and Image J. analyzed the gray value. Set up 3 groups to repeat the experiment. The key antibodies included: Nephrin (No. 503048, ZenbioN, China, 1:3000); β-actin (No. ab822682, Abcam, UK,1:1000); cleaved caspase1 (No.341030, ZenbioN, China,1:2000); Caspase3 (No.AF6311, Affinity, China,1:2000); IL-18 (No.R24693, ZenbioN, China,1:800); Anti-Integrin beta 8 (No.ab243023, Abcam, UK,1:1000); cleaved caspase-3 (No.9664,CST, China,1:1000); Caspase-1 (No. 83383,CST, China,1:2000).

#### Statistical processing

The WB grayscale data is presented in the form of mean ± SD. Statistical analyses were performed using one-way ANOVA with multiple comparisons and two‐tailed Student’s t tests. Value was considered to be statistically significant when *P < 0.05, **P < 0.01 and ***P < 0.001.

## Results

### Network pharmacology to identify potential inflammatory-related drug targets for NGR1 and DN

**Fig. 1:**
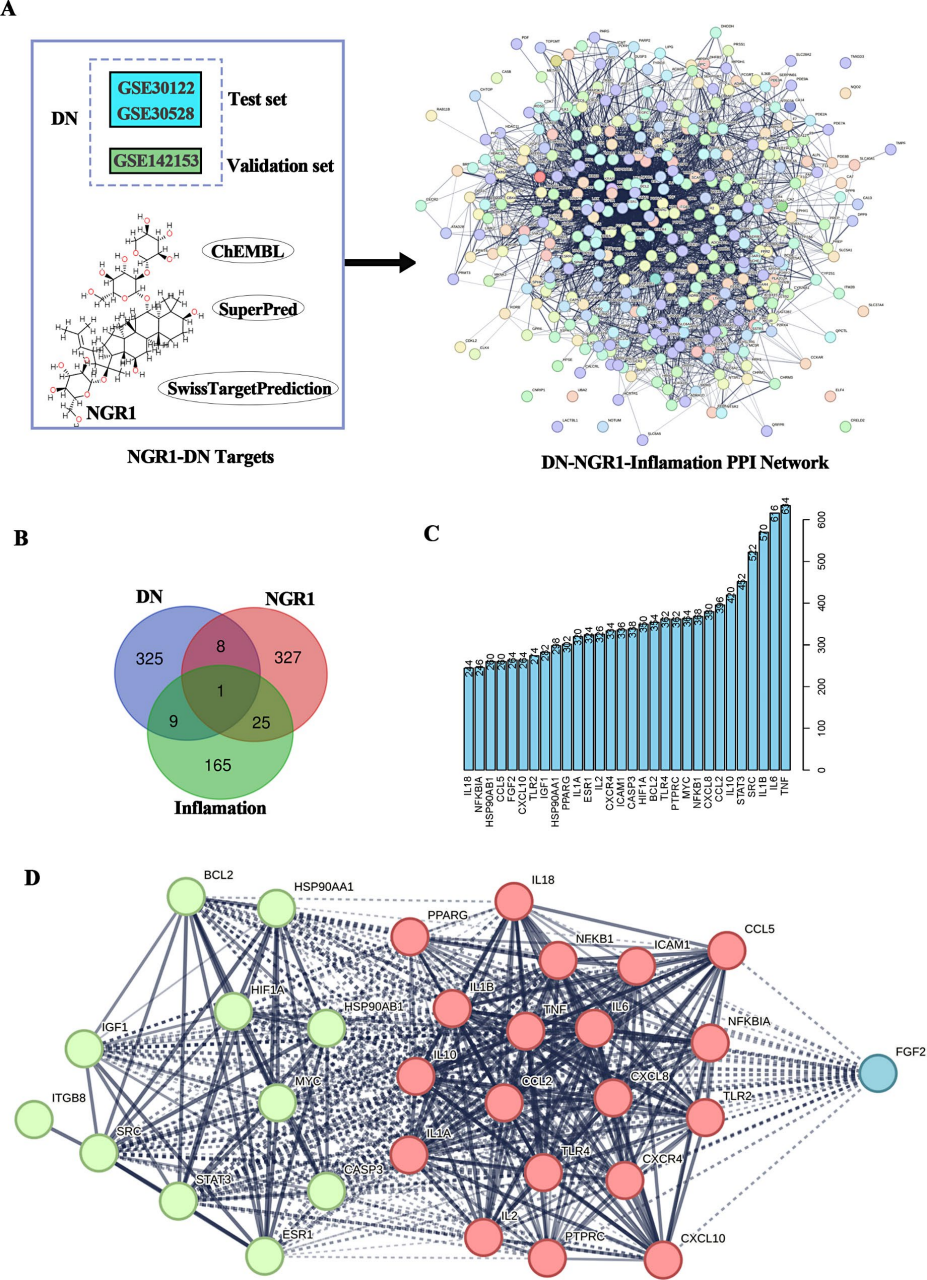
Pharmacological analysis of NGR1 and inflammatory target network of DN. **A** The transcriptome data of DN patients were analyzed by Limma package, including GSE30122, GSE30528 and GSE142153. The potential drug targets of NGR1 were identified using three approaches: ChEMBL, SuperPred, and Swiss Target Prediction; **B** Disease and drug interaction network of DN and NGR1; **C** inflammation-associated proteins for NGR1 treatment of DN; **D** Network of interactions between NGR1 inflammatory regulatory drug targets and DN disease

As a chronic microangiopathy disease, DN plays a key role in the regulation of inflammation. In this study, the transcriptome data of DN patients were analyzed by Limma package, including GSE30122, GSE30528 and GSE142153. The potential drug targets of NGR1 were identified using three approaches: ChEMBL, SuperPred, and Swiss Target Prediction (Fig. 1A); we constructed a disease and drug interaction network of DN and NGR1 (Fig. 1B); some inflammation-associated proteins were found to be potential drug targets for NGR1 treatment of DN (Fig. 1B, C); we constructed a network of interactions between NGR1 inflammatory regulatory drug targets and DN disease (Fig. 1D); The results showed that ITGB8 protein was the key regulatory protein of NGR1 inflammation-related drugs, and it could be potentially associated with Caspase 3, IL-18 and other proteins.

### NGR1 inflammatory drug target ITGB8 molecular docking verification and molecular dynamics simulation experiments

**Fig. 2:**
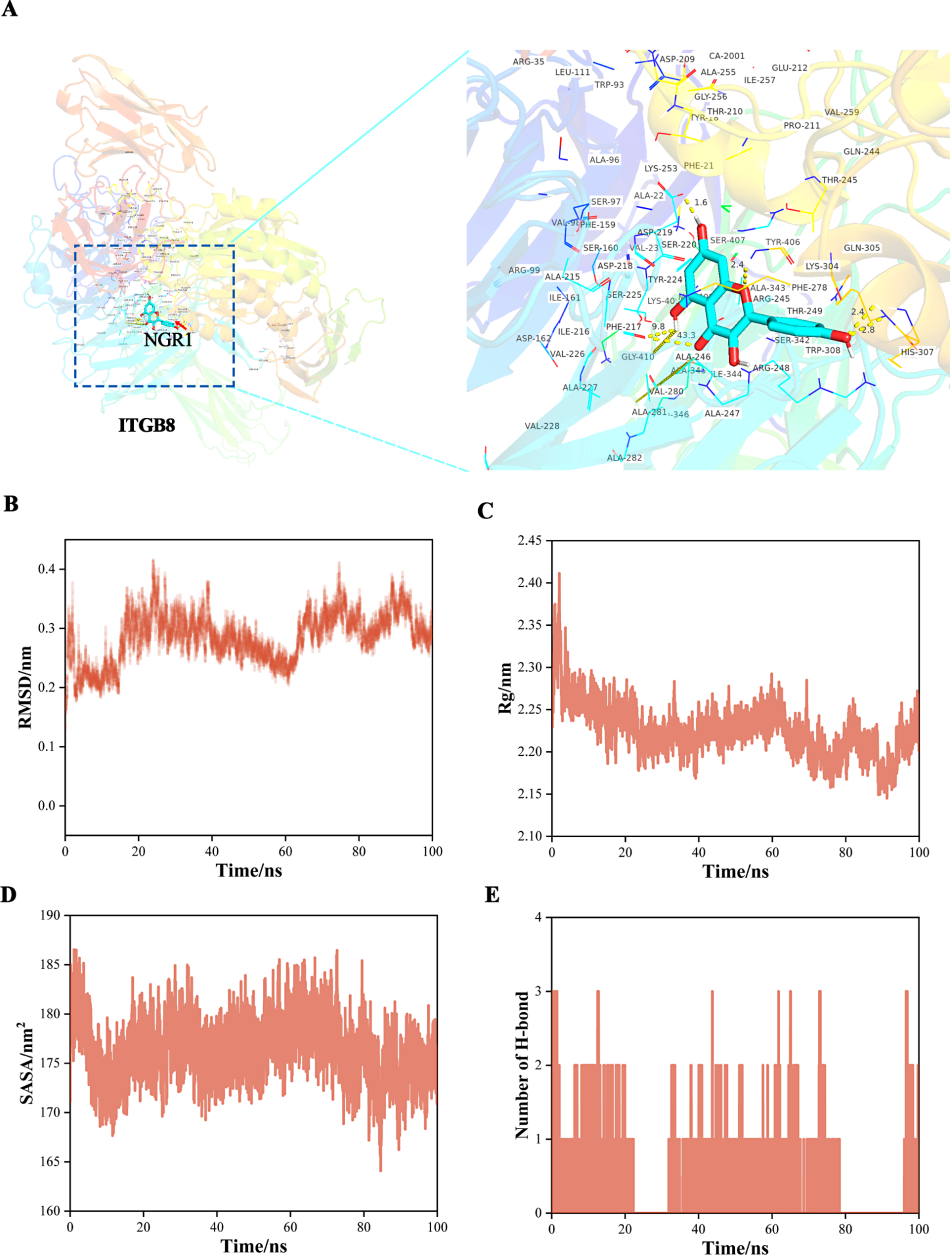
Molecular docking verification and Molecular Dynamics Simulation Experiments validates the interaction between NGR1 and ITGB8. **A** molecular docking validates the interaction between NGR1 and ITGB8; **B** Molecular Dynamics Simulation Experiments validates the interaction between NGR1 and ITGB8

Network pharmacological methods identified ITGB8 as a potential drug target of DN. We used molecular docking to further screen the pharmacodynamic groups between drugs and diseases. Hydrogen bonds were observed between the hydroxyl group of SER-407 and the backbone carbonyl group of ALA-22; Hydrogen bonds were identified between the amide nitrogen of ALA-343 and the hydroxyl group of TYR-406.The docking results revealed several critical hydrogen bond interactions that suggest a stable binding of the ligands to the ITGB8 protein. These interactions indicate that the ligands can effectively bind to ITGB8, supporting its potential as a therapeutic target for DN. The detailed binding interactions provide insights into the molecular mechanisms underlying the pharmacodynamic effects of the drugs, guiding the rational design of more effective therapeutic agents (Fig. 2A); At the molecular level, we have employed molecular dynamics simulations to unequivocally demonstrate the interaction between NGR1 and ITGB8. Key highlights from these simulations include: RMSD Analysis: The stable RMSD values, fluctuating narrowly between 0.1 and 0.2 nm after 20 nanoseconds (ns), signify a robust and stable ligand-protein complex structure. Rg Analysis: The minimal fluctuations in the radius of gyration (Rg), consistently maintained within 2.2–2.3 nm, indicate that the overall protein size remains unchanged, with no significant structural alterations.Hydrogen Bond Analysis: The stabilization of hydrogen bond counts at 3–4 bonds after 40 ns underscores the robust and stable interaction between the ligand and protein. SASA Analysis: The relatively constant solvent-accessible surface area (SASA) values (175–180 nm²), reflect a balanced and stable interaction between the complex and the solvent. MM-PBSA Binding Energy Decomposition: The MM-PBSA calculation reveals a substantial binding affinity between the ligand and protein, with a binding free energy of -6.54 ± 15.18 KJ/mol. Notably, polar solvation free energy (EPB) and van der Waals interaction energy (EVdw) are the primary driving forces, while Coulombic energy (ECoul) and solvent-accessible surface area (ESASA) also contribute significantly to the overall binding affinity (Fig. 2B).

### ITGB8 can indicate the progression of renal function

**Fig. 3:**
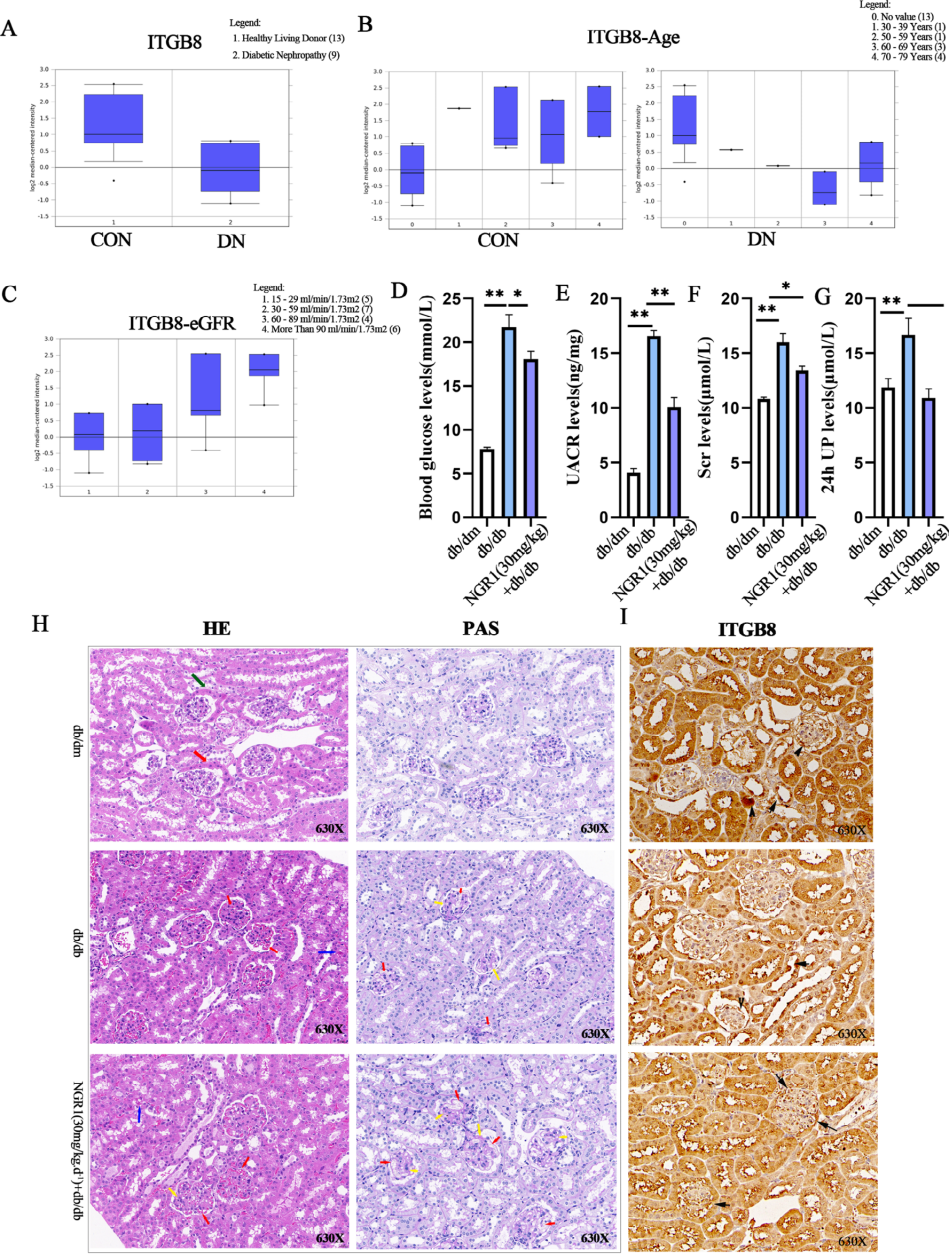
Nephroseq database and db/db model set up to analyses the clinical correlation between ITGB8 and DN. **A** Difference in ITGB8 expression between DN and normal controls; **B** Effect of age on ITGB8 expression; **C** Expression of ITGB8 at different stages of eGFR; **D**-**G** 10 days after drug administration, blood was collected from the tail vein for the detection of blood glucose, urine albumin-to-creatinine ratio (UACR), creatinine, and 24-hour urine albumin; **H** Pathological examinations included PAS staining and HE staining; **I** To construct a DN model, db/db mice (*n* = 3) were used, with db/dm littermates (*n* = 3) at 8 weeks old serving as controls. The NGR1 intervention group (*n* = 3) was established based on the db/db mouse model, where NGR1 (30 mg/kg) was administered via intraperitoneal injection for 10 consecutive days starting from the 8th week. The expression of ITGB8 was subsequently evaluated. * *P* < 0.05 , **P <0.01 , ***P <0.001

437 RNA-Seq samples have been added to Nephroseq Classic (V4). We used Nephroseq database to obtain DN and healthy control sequencing samples to analyze ITGB8-related clinical value. The transcriptome-level level of ITGB8 was found to be lower in the DN group than in the control group (Fig. 3A); among patients with DN at different age stages, ITGB8 expression was downregulated in the 50 to 80-year-old compared with normal peers (Fig. 3B); eGFR expression was lowest at levels of 15–29 ml-min 1.73 m^2^, when patients were in the kidney failure phase (Fig. 3C). 10 days after drug administration, blood was collected from the tail vein for the detection of blood glucose, urine albumin-to-creatinine ratio (UACR), creatinine, and 24-hour urine albumin. The results revealed that NGR1 (30 mg/kg) intervention significantly improved and reduced the levels of blood glucose, UACR, Scr, and 24-hour urine albumin (Fig. 3D-G). Observations revealed significant morphological changes in the kidneys of mice in various groups. Compared with the db/m control group, the db/db disease model group exhibited mesangial cell proliferation and increased mesangial matrix, while NGR1 (30 mg/kg) treatment significantly reduced mesangial cell proliferation, decreased mesangial matrix expansion, and capillary dilation in db/db mice, with statistically significant differences (Fig. 3H). Immunohistochemical staining was used to evaluate the expression of ITGB8 in the kidneys of db/db mice after 10 consecutive days of intraperitoneal injection of NGR1 (30 mg/kg). The expression of ITGB8 in the glomerular basement membrane region was significantly increased in the db/db group compared with the control (*P* < 0.05) (Fig. 3I).

### NGR1 can attenuate podocyte injury induced by high glucose

**Fig. 4:**
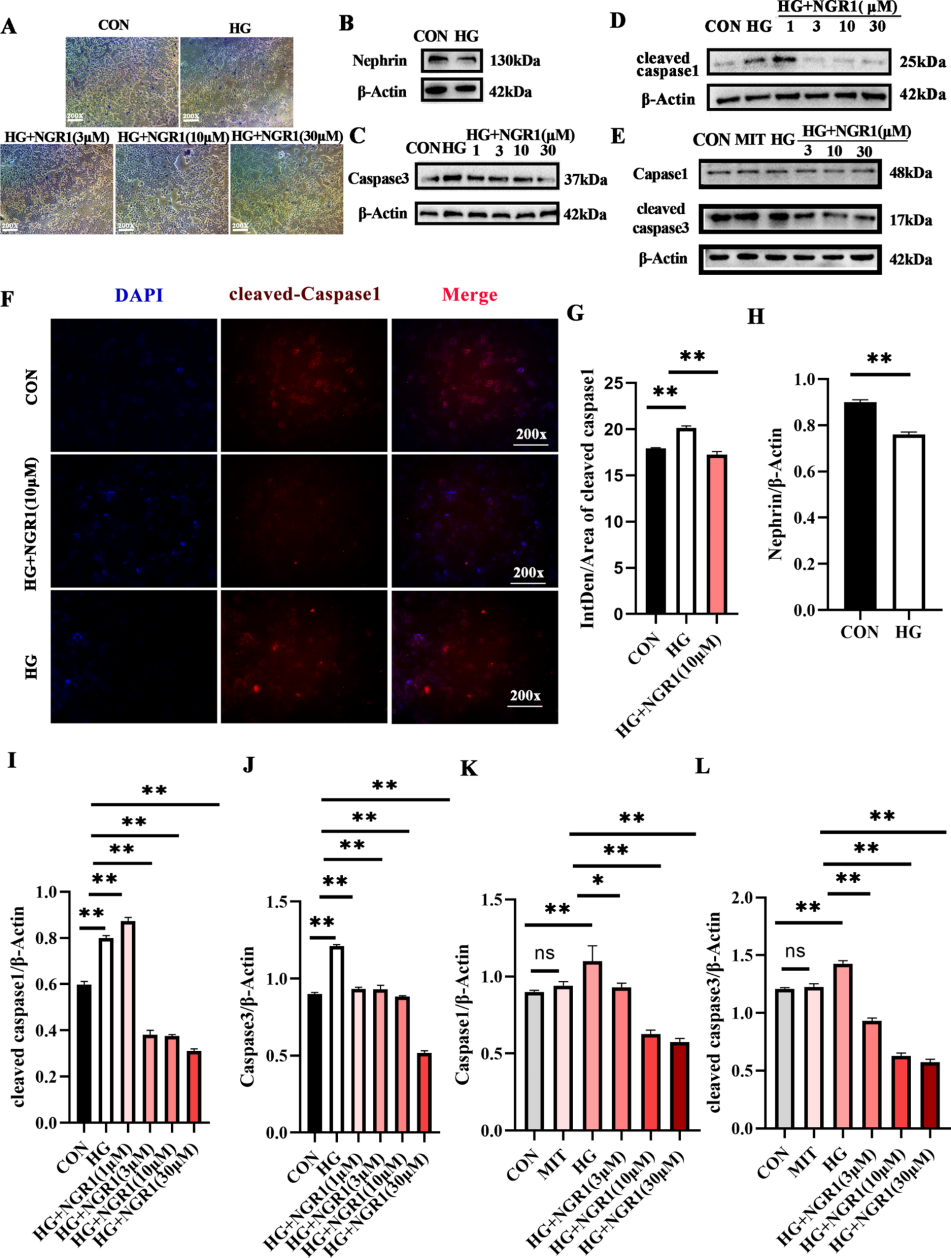
NGR1 can slow podocyte apoptosis and reduce podocyte pyrogen under high glucose. **A** Substantial reduction in the number of necrotic cells was observed; **B**-**D** The expressions of Nephrin, cleaved caspase1 and Caspase3 by WB analysis, group as CON, HG (30 mM), HG (30 mM) + NGR1(1, 3, 10, 30 µM); **E** The expressions of Nephrin, cleaved caspase3 and Caspase1 by WB analysis, group as CON, MIT(30 mM), HG (30 mM), HG (30 mM) + NGR1(3, 10, 30 µM); **F**-**G** Immunofluorescence detection of cleaved caspase1 expression and statistical analysis; **H**-**L** one-way ANOVA with multiple comparisons and two‐tailed Student’s t tests of protein expression each group was replicated in three separate experiments. * *P* < 0.05, * * *P* < 0.01,***P < 0.001

We employed trypan blue as a marker to identify necrotic cells and observed that the percentage of necrotic cells in the high-glucose treatment group exceeded that of the normal control group. Notably, following the introduction of NGR1 intervention, a substantial reduction in the number of necrotic cells was observed (Fig. 4A). Further analysis revealed that the expressions of Nephrin and Caspase3 were conspicuously downregulated in the high-glucose group compared to the normal control (Fig. 4B, H and J). Remarkably, subsequent intervention with NGR1 drugs at varying concentrations (1, 3, 10, 30 µM) led to a significant decrease in Caspase3 expression. Additionally, we discovered a substantial upregulation in the expression of cleaved caspase1 in the high-glucose group, which was effectively suppressed following the administration of NGR1 (1, 3, 10, 30 µM) (Fig. 4D and I), the expression of cleaved caspase1 can be inhibited by NGR1 (Fig. 4F and G). Meanwhile, we observed that the expressions of caspase1 and Cleaved caspase3 were significantly up-regulated in high glucose state and significantly down-regulated after drug intervention (Fig. 4E, K and L).

These findings indicate the potential therapeutic effects of NGR1 in modulating cell death pathways associated with high-glucose conditions.

### NGR1 can inhibit inflammation and alleviate podocyte injury

**Fig. 5:**
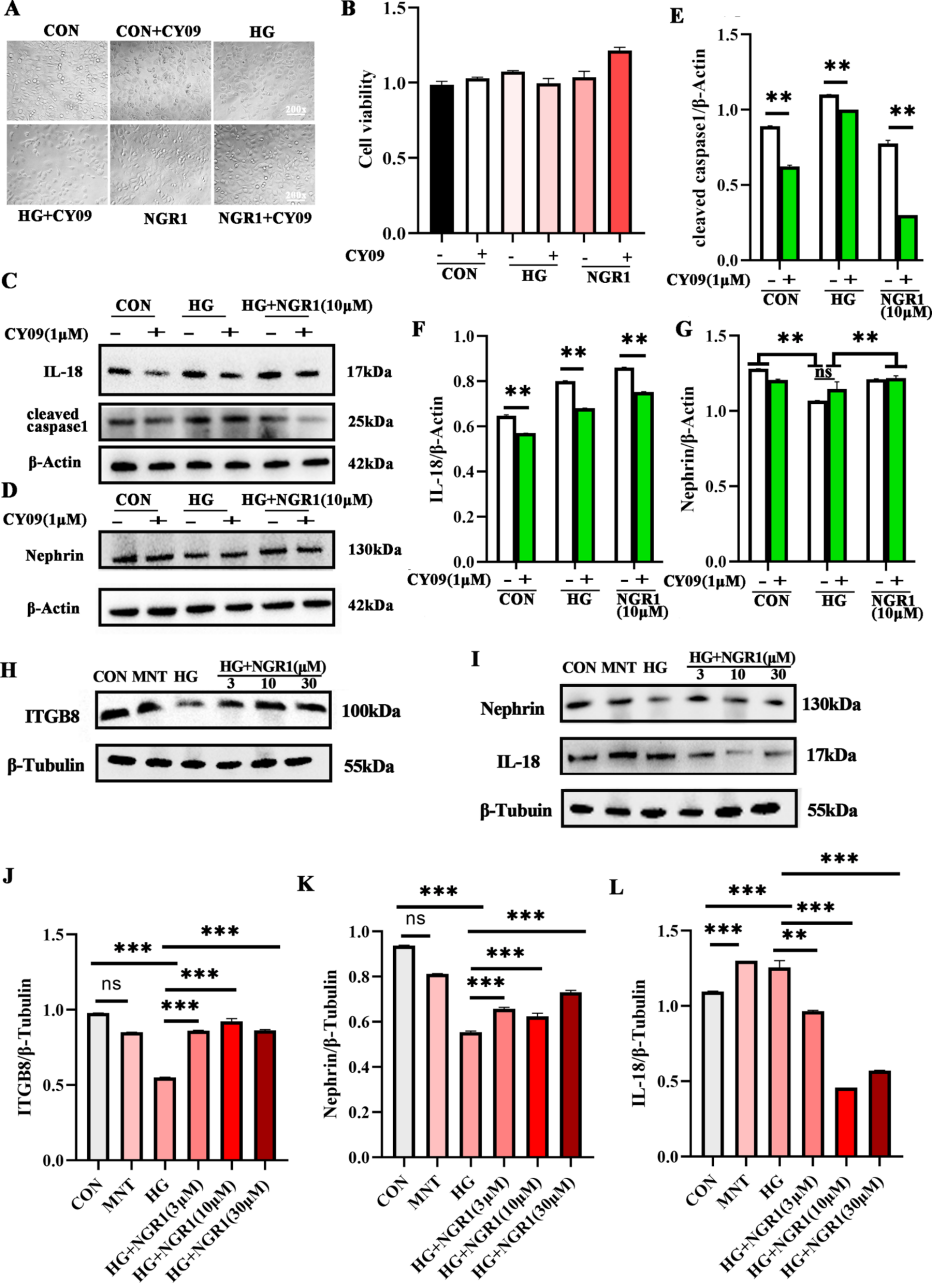
NGR1 relieves podocyte injury by inhibiting the inflammation. **A** Using cytotoxicity assay (CCK8) to evaluate the effect of NLRP3 inhibitors (CY09 1μM) on podocyte activity; **B** statistical analysis of cleaved caspase1; **C**, **D** Western blot analysis of cleaved caspase1, IL-18 and Nephrin; **E**-**G** one-way ANOVA with multiple comparisons and two‐tailed Student’s t tests of Nephrin, IL-18 and cleaved caspase1 protein expression; **H, I**: Western blot analysis of ITGB8, Nephrin and IL-18 in NGR1protect funcation to DN. * *P* < 0.05, * * *P* < 0.01,***P < 0.001. Each group was replicated in three separate experiments

**Fig. 6:**
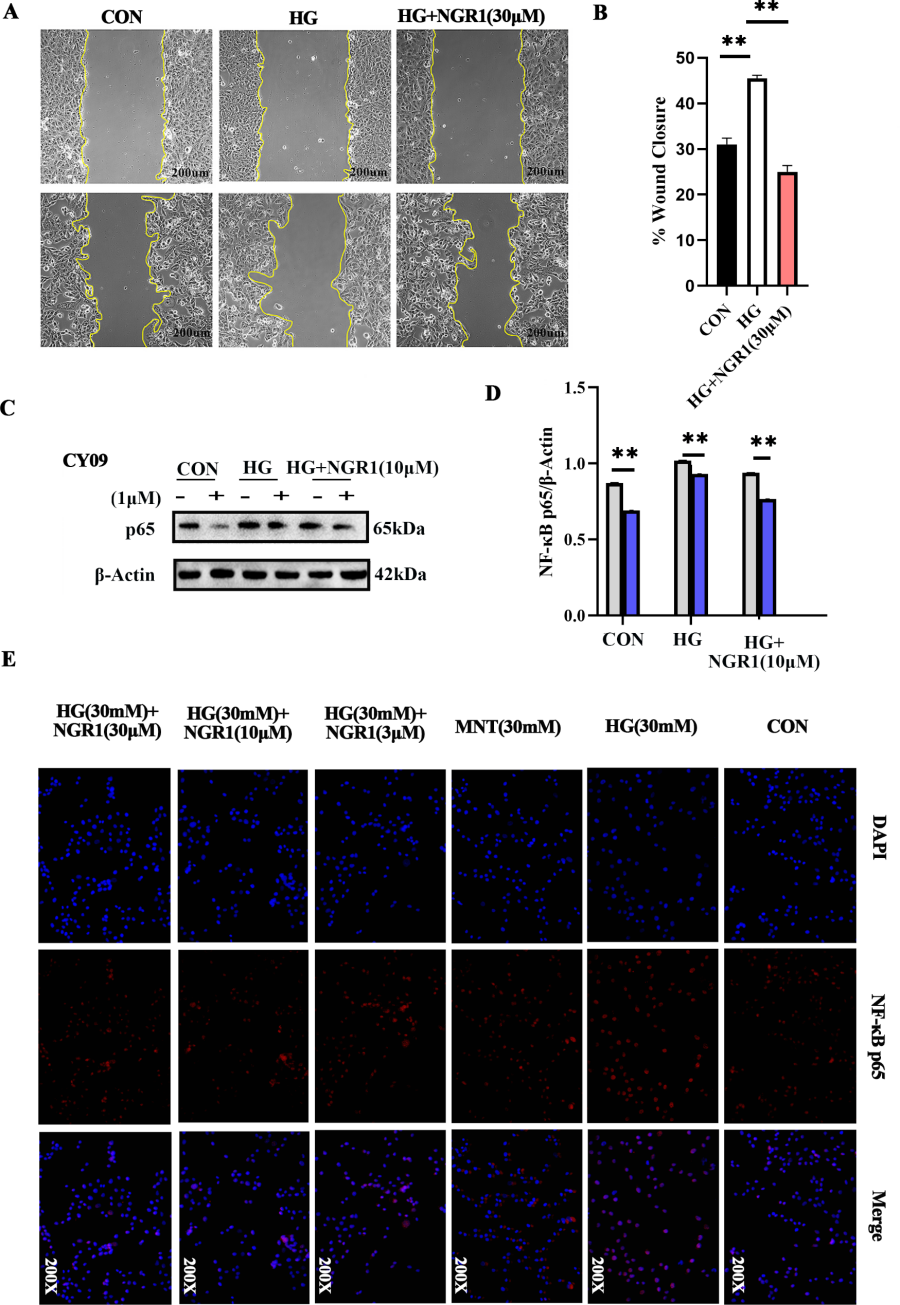
Effects of high glucose and therapeutic interventions on podocyte migration and NFκB p65 expression. **A**, **B** This graph depicts the ability of podocytes to repair a scratch area under different experimental conditions. **C**,**D** NFκB p65 expression levels following CY09 treatment, and displays the expression levels after NGR1 intervention; **E** The NF-κB detection reagent to evaluated the NGR1 protect funcation to DN. Each group was replicated in three separate experiments.*P <0.05,**P <0.01, ***P <0.001

Utilizing the CCK8 assay to evaluate podocyte viability across various groups, we observed no significant difference in cellular activity between them (Fig. 5A). Notably, the expressions of Nephrin and cleaved caspase1 were substantially elevated in the high-glucose group compared to the normal control (Fig. 5B, C). Interestingly, upon intervention with NGR1 at varying concentrations (1, 3, 10, 30 µM), the expression of cleaved caspase1 was significantly reduced. Furthermore, under the condition of NLRP3 inhibitor CY09 intervention, both Nephrin and cleaved caspase1 expressions were significantly downregulated (Fig. 5C-G). These findings provide insights into the regulatory mechanisms of podocyte function under high-glucose conditions and the potential therapeutic effects of NGR1 and NLRP3 inhibitors. Besides, we observed the expressions of Nephrin, IL-18 and ITGB8 were substantially elevated in the high-glucose NGR1 at varying concentrations (3, 10, 30 µM) and MNT group compared to the normal control (Fig. 5H and I). Interestingly, upon intervention with NGR1 at varying concentrations (3, 10, 30 µM), the expression of IL-18 was significantly reduced. Furthermore, both Nephrin and ITGB8 expressions were significantly downregulated (Fig. 5J-L). These findings provide insights into the regulatory mechanisms of podocyte function under high-glucose conditions and the potential therapeutic effects of NGR1. In the control group, podocytes were able to significantly repair the scratched area within 24 h; whereas in the high-glucose treatment group, only a partial repair of the scratched area was observed during the same time frame. After NGR1 intervention, the migration of podocytes was promoted, significantly reducing the scratched area. (Figure 6A and B). under the condition of NLRP3 inhibitor CY09 and NGR1 intervention, both NFκB p65 expressions were significantly downregulated (Fig. 6C, D), The detection of NFκB p65 nuclear translocation ability revealed that the expression of NFκB p65 in the nucleus of the HG group increased compared with that in the normal control group. After intervention with different concentrations of NGR1 (3, 10, 30 µM), the nuclear translocation ability of NFκB p65 was inhibited (Fig. 6E).

## Discussion

We used ChEMBL, SuperPred, and Swiss Target Prediction network pharmacology methods to find possible drug targets linked to NGR1 and DN. We discovered that the ITGB8 protein is a key regulatory protein for NGR1’s drugs that target inflammation. It does this by setting up a network of interactions between the DN and the drug targets of NGR1’s moderating inflammation. This protein may have associations with caspase 3, IL-18, and other proteins. Molecular docking techniques showed that pharmacodynamic targets like SER-407 and ALA-22, as well as Ala-343 and TYR-406, have hydrogen bond interactions in different three-dimensional models of NGR1 and ITGB8. Using the Nephroseq database, we conducted a thorough examination of the clinical significance of ITGB8. The study revealed that the transcriptome level of ITGB8 was lower in the DN group compared to the control group. Among DN patients in different age groups, ITGB8 expression was found to be downregulated in the 50–80 age group when compared to their normal counterparts. Additionally, eGFR expression was found to be at its lowest level (15–29 ml/min, 1.73 m^2^), indicating kidney failure. In our study, db/m mice displayed normal rodent kidney tissue without significant pathological changes. In the db/db group, PAS-stained sections revealed moderate microvascular dilation within the glomerulus, basement membrane thickening, and occasional tubular epithelial cell degeneration. In the NGR1 (30 mg/kg) group, the extent of basement membrane thickening was reduced. This effect might be partially attributed to the anti-inflammatory properties of NGR1, although the exact mechanism remains unclear. In the db/db mouse model, downregulation of ITGB8 expression in renal tissue was associated with renal inflammatory damage. Simultaneously, fundamental research has validated that NGR1 has the ability to impede inflammatory reactions and diminish podocyte damage. A clear sign of DN is a drop in the number of glomerular cells due to inflammatory responses, programmed cell death, and the buildup of extracellular matrix.

Integrins are heterodimeric receptors that regulate cell adhesion, migration, and apoptosis. Integrin αvβ8 is most abundantly expressed in the kidney and brain, with latent transforming growth factor-β (TGF-β) being its primary ligand. In the kidney, αvβ8 is localized in mesangial cells, which are adjacent to glomerular endothelial cells and maintain the structure of glomerular capillaries through mechanical and paracrine mechanisms that are not yet fully understood. Research has elucidated a unique communication mechanism between mesangial and endothelial cells, wherein mesangial αvβ8 maintains glomerular microvascular integrity by sequestering TGF-β in a latent form. In pathological conditions associated with reduced αvβ8 expression and increased TGF-β secretion, the compensatory regulation of PECAM-1 contributes to the survival of glomerular endothelial cells (Khan et al. 2011). When αvβ8 expression is reduced or its function impaired, latent TGF-β may be released and activated, stimulating pathological changes such as apoptosis in glomerular endothelial cells. This pathological process is closely linked to the development and progression of various kidney diseases, including glomerulonephritis and DN.

Our study found that in the db/db mouse model, downregulation of ITGB8 expression in renal tissue was associated with renal inflammatory damage. ITGB8 plays a critical role in maintaining glomerular microvascular integrity and regulating TGF-β signaling. Its downregulation may lead to excessive activation of TGF-β, thereby promoting renal fibrosis and loss of function. Further experiments revealed that NGR1’s regulation of ITGB8 might be closely associated with the inhibition of NF-κB nuclear translocation and NLRP3-mediated inflammatory signaling activation. Additionally, decreased ITGB8 expression may increase the risk of glomerular endothelial cell apoptosis, further exacerbating the pathological process of DN. Endothelial cell damage not only impairs glomerular filtration function but also promotes inflammation and fibrosis. In an assessment of apoptosis levels in human renal podocytes under high glucose conditions, we found that hyperglycemia induced partial podocyte apoptosis. Through integrated drug target prediction and molecular dynamics simulation validation, we discovered that NGR1 could stably bind to ITGB8. However, further research is needed to confirm this finding through CESTA, DARTS, and other experimental methods.

Hyperglycemic conditions trigger chronic vascular inflammation, which results in local tissue damage in the glomerulus (Bayaraa et al. 2022). The damaged tissue undergoes fibrotic repair, leading to glomerulosclerosis and eventually causing kidney dysfunction. This state of affairs is highly regrettable for management of DN (Huang et al. 2023). During the progression of DN, there is an increase in the size of podocytes, leading to a decrease in the presence of surface adhesion proteins like nephrin (Fu et al. 2020). Besides, the filtration membrane barrier is exposed to a high blood sugar environment. The accumulation of sugars and proteins in the glomerulus puts additional strain on the glomerular filtration process, resulting in a reduction in the rate at which the kidneys filter waste products. This leads to glomerular hyperfiltration, increased blood flow to the glomerulus, and hypertension (Zuo et al. 2024; He et al. 2020). Damage to barrier tissues initiates an inflammatory reaction within the body. In the glomeruli of rats with DN, NGR1 increases the expression of renin and podocin. This suggests that NGR1 protects podocytes by managing the expression of nephrin and podocin (Niec et al. 2021). This effect may be linked to inflammation regulation, although the exact mechanism is not yet understood.

During diabetic stress, cells in the kidneys cause proinflammatory reactions. These reactions activate the body’s innate immune system by releasing chemokines, cell adhesion molecules, and damage-associated molecular patterns (DAMPs), which then attract macrophages (Chen et al. 2022). It improves the innate immune response by starting the complement system and bringing in cytokines. This makes it easier for inflammatory cells and mast cells to get into the renal tissue (Rawish et al. 2021). NF-κB is often activated in various proinflammatory responses(Liu et al. 2017). Different signals activate NF-κB, which breaks down the inhibitor of NF-κB kinase (IKK). This lets activated NF-κB get into the nucleus and attach to DNA, which starts the transcription of genes that cause inflammation, like cytokines, chemokines, leukocyte adhesion molecules, cell receptors, and growth factors (Dorrington and Fraser 2019). These findings indicate that immune and inflammatory regulation play a significant role in the development and progression of DN.

The level of integrin subunit beta 8 (ITGB8) has been found to rise in microglia, which lowers inflammation and cell death through its own mechanism (Bai and Niu 2020). It was found that SER-407 and ALA-22, as well as Ala-343 and TYR-406, interact with pharmacodynamic targets through hydrogen bonds in different three-dimensional models of NGR1 and ITGB8. These interactions can reduce the speed of inflammatory responses when there is excessive glucose in the blood, thereby providing protection to podocytes. Based on these results, it looks like the way NGR1 improves DN is closely connected to the inflammatory signals that come from ITGB8.In the end, this study used a variety of pharmacological methods to find possible drug targets for NGR1 and DN, focusing on the ITGB8 protein. Hydrogen bond interactions between NGR1 and ITGB8 were identified using molecular docking techniques. These interactions have the potential to inhibit inflammatory responses and provide protection to podocytes in hyperglycemic conditions. The reduced expression of ITGB8 in patients with DN indicates its importance in the disease’s advancement. These discoveries provide novel pharmaceutical targets and avenues for research into DN treatment.

Mesangial proliferation is a significant feature in the pathological process of DN and is closely associated with glomerulosclerosis and declining renal function. In diabetes, hemodynamic changes and the abnormal release of vasoactive substances induced by hyperglycemia can cause microvascular dilation within the renal glomerulus (Dong and Tang 2022). This dilation may increase glomerular filtration pressure, further leading to kidney damage. The basement membrane is a crucial component of the glomerular filtration barrier. Under diabetic conditions, factors such as hyperglycemia, oxidative stress, and inflammation may lead to basement membrane thickening (Zhao et al. 2022). Such thickening can impair glomerular filtration, resulting in manifestations of kidney damage, such as proteinuria.

In clinical validation, compared with healthy controls or with increasing age, transcriptomic data from the renal tissues of DN patients showed a significant downregulation of ITGB8 expression, suggesting that ITGB8 could serve as a valuable indicator of renal insufficiency. This finding is consistent with observations in the db/db mouse model, further confirming ITGB8’s potential role in the pathogenesis of DN. Our animal experiments indicated that NGR1 might help restore or enhance ITGB8 function in the kidney by upregulating ITGB8 expression. This includes maintaining glomerular microvascular integrity, inhibiting excessive NF-κB activation, and protecting glomerular podocytes. These combined effects on the kidney contribute to slowing the progression of DN and improving patient prognosis.

Given the critical roles of NGR1 and ITGB8 in DN, developing therapies targeting these molecules holds significant potential. For example, upregulating NGR1 and ITGB8 expression or activity through drugs or biologics could represent a novel therapeutic strategy for DN. This approach may offer better efficacy and fewer side effects, providing DN patients with improved treatment options.

## Conclusion

In summary, NGR1 exhibits great potential in the treatment of DN by mitigating renal mesangial proliferation and reducing kidney damage, as well as upregulating ITGB8 expression. Future research should further explore the specific mechanisms by which NGR1 and ITGB8 contribute to the pathogenesis of DN and accelerate the development of therapies targeting these molecules to provide DN patients with more effective and safer treatment options.

## Data Availability

No datasets were generated or analysed during the current study.

## Abbreviations

Caspase-1: L-Aspartic Acid Protease-1
DN: Diabetic nephropathy
DM: Diabetes mellitus
HPCC: Human primary podocytes
NGR1: Notoginsenoside R1
NLRP3: Domain samples and receptor-related protein 3
NF-κB: Nuclear factor kappa B
TCM: Traditional chinese medicine
